# Sonic hedgehog expression in the postnatal brain

**DOI:** 10.1242/bio.040592

**Published:** 2019-03-05

**Authors:** Aileen Rivell, Ronald S. Petralia, Ya-Xian Wang, Ellie Clawson, Keelin Moehl, Mark P. Mattson, Pamela J. Yao

**Affiliations:** 1 Laboratory of Neurosciences, NIA/NIH, Baltimore, MD 21224, USA; 2 Advanced Imaging Core, NIDCD/NIH, Bethesda, MD 20892, USA; 3 Department of Neuroscience, Johns Hopkins University School of Medicine, Baltimore, MD 21205, USA

**Keywords:** Sonic hedgehog, Endogenous, Hippocampus, Neuron

## Abstract

Beyond its role in patterning the neural tube during embryogenesis, additional functions of Sonic hedgehog (Shh) in post-embryonic and mature brains have been coming into focus. However, the question of the abundance of endogenous Shh – the ligand of the signaling pathway – and its changes over time in post-embryonic and mature brains are less well understood. Here we find that while the amounts of Shh transcript and protein in rat brains are nearly undetectable at birth, they increase continuously during postnatal development and remain at readily detectable levels in young adults. This developmental age-associated increase in Shh levels is also seen in hippocampal neurons grown in culture, in which very young neurons produce minimal amounts of Shh protein but, as neurons grow and form synapses, the amounts of Shh increase significantly. Using immunolabeling with antibodies to different residues of Shh, we observed that the N-terminal fragment and the C-terminal fragment of Shh are present in hippocampal neurons, and that these two Shh forms co-exist in most compartments of the neuron. Our findings provide a better understanding of Shh expression in the brain, laying the groundwork for further comprehending the biogenesis of Shh protein in the young and mature brain and neurons.

## Abstract (precis)

**Summary:** Endogenous Sonic hedgehog protein in the hippocampus is undetectable at birth but increases continuously during postnatal development and remains at readily detectable levels in the mature brain.

## INTRODUCTION

The Sonic hedgehog (Shh) signaling pathway regulates diverse cellular processes (Ingham and McMahon, 2001). In the nervous system, besides its best-characterized role in patterning the embryonic neural tube (Chiang et al., 1996; Dessaud et al., 2008), there is accumulating evidence that Shh signaling continues to exist post-embryonically and carries out a range of functions. For example, Shh is known as a mitogen for different populations of neural precursors or progenitor cells (Wechsler-Reya and Scott, 1999; Lai et al., 2003; Palma et al., 2005; Han et al., 2008). Shh also controls specific properties of astrocytes in the brain (Garcia et al., 2010; Farmer et al., 2016). Furthermore, Shh has been found to be important for the growth of axons in several types of neurons including spinal cord commissural neurons (Charron et al., 2003; Parra and Zou, 2010), midbrain dopaminergic neurons (Hammond et al., 2009), and hippocampal neurons (Yao et al., 2015). In the case of hippocampal neurons, Shh-stimulated axonal growth is accompanied by elevated Gli1 expression (Yao et al., 2015), enhanced autophagy (Petralia et al., 2013), and activated mitochondria (Yao et al., 2017). Despite the abiding interest in what roles Shh plays in the nervous system, limited attention has been given to the source and biogenesis of Shh itself, particularly the endogenous Shh levels in the natural non-genetically modified brain. In this study, we have systematically examined Shh levels in the brain at different ages from embryos to young adults and have established a temporal expression of Shh. Our analysis reveals appreciable amounts of Shh in postnatal and young adult brains, including in the hippocampus.

## RESULTS

### Endogenous Shh expression levels in the rat brain during embryonic and postnatal development

**Fig. 1.**
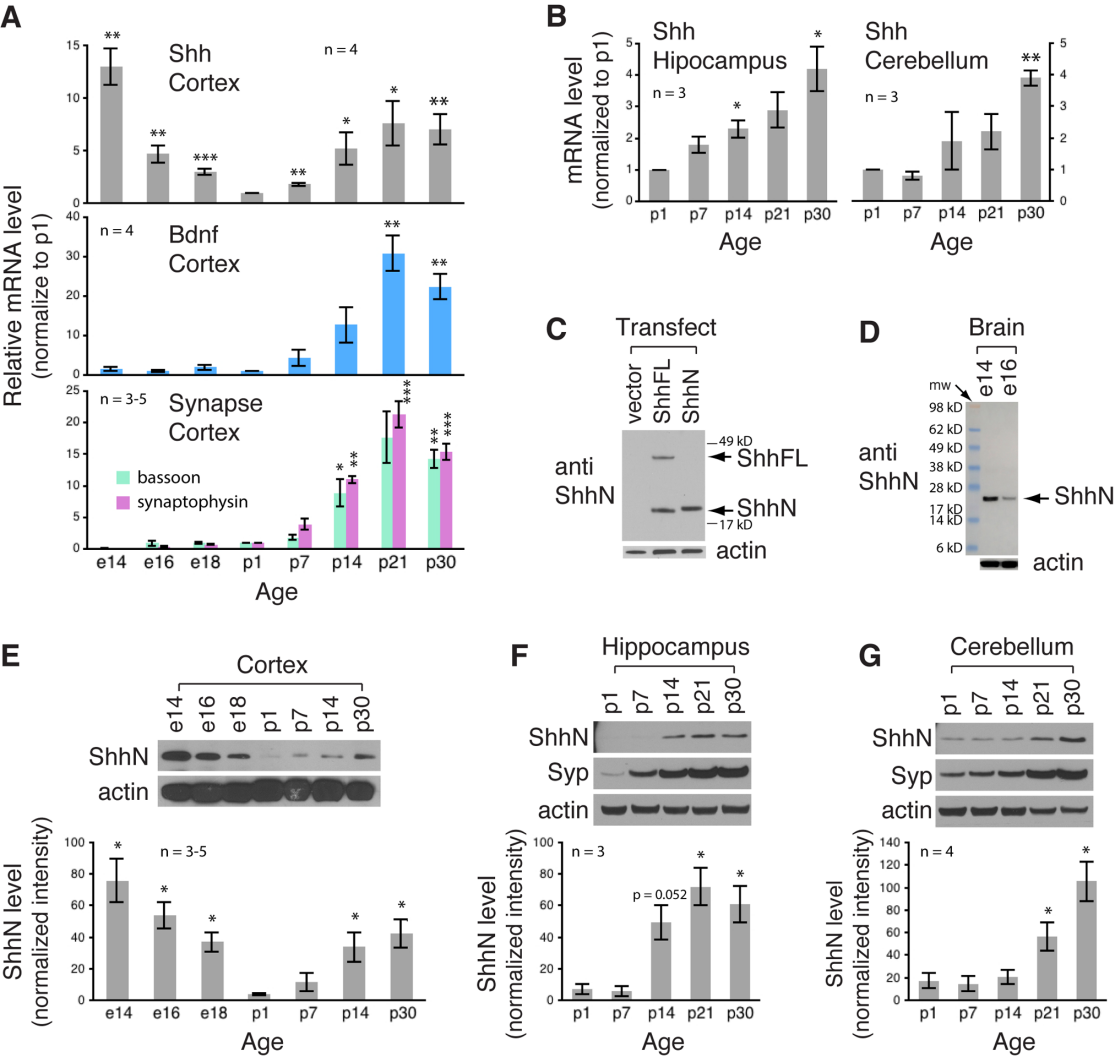
**Shh mRNA and ShhN protein expression levels in rat brains during embryonic and postnatal development.** (A) Quantitative RT-PCR assay of Shh mRNA level in rat cortex from embryonic day 14 (e14) to postnatal day 30 (p30). For comparison, mRNA levels of Bdnf and synaptic markers, bassoon and synaptophysin, were also assayed. The data were expressed as fold change from p1 and plotted against age (*n*=the number of rat embryos or pups per age). For e14 embryos, whole brains were used. Data of quantitative RT-PCR using a different Shh primer set are shown in Fig. S1A. (B) Shh mRNA level in postnatal rat hippocampus and cerebellum from p1 to p30. (C) Immunoblot with an antibody to N-terminal epitope of Shh. Lanes contain lysates from HEK cells transfected with wild-type full-length Shh (ShhFL), N-terminal fragment of Shh (ShhN), or vector. The ShhN antibody detects N-terminal fragment (∼19 kD) and full-length (∼45 kD) Shh. (D) Immunoblot with the ShhN antibody showing the ∼19 kD ShhN as the main Shh species in tissue extracts from e14 or e16 rat brains. A similar finding from mouse brain extracts using this antibody is shown in Fig. S1B. Also, a similar finding from rat brain extracts but using a different ShhN antibody is shown in Fig. S1C. (E) Immunoblot analysis of ShhN protein level in rat cortex from embryonic day 14 (e14) to postnatal day 30 (p30). ShhN level is high during embryonic development, declines to a nearly undetectable level at birth, and increases again during late postnatal development. Blot shown is one representative experiment; blots of additional experiments are shown in Fig. S1D. Histogram includes at least three experiments. (F,G) Immunoblot analysis showing that, similar to cortex, ShhN protein level in hippocampus (F) and cerebellum (G) increases during late postnatal development. Syp, synaptophysin. Error bars represent s.e.m. ***P<0.001, **P<0.01, *P<0.05, unpaired *t*-test. The values of ShhN protein level represent the ShhN band intensities normalized to the actin band intensities.

Starting with Shh mRNA, we measured its expression levels in the rat cortex from embryonic day 14 (e14) to postnatal day 30 (p30) using quantitative RT-PCR. Analysis of four biological replicates (number of embryos or pups) revealed that Shh mRNA level in the cortex was high at e14, but declined gradually as embryos developed, and became undetectable at birth (top graph in Fig. 1A). Notably, after birth and throughout postnatal development, Shh mRNA became detectable again and its level increased steadily as young rats matured, and reached a plateau between p21 and p30 (p21 versus p1, 7.6±2.1 versus 1, *P*=0.0259; p30 versus p1, 7.0±1.4 versus 1, *P*=0.0084; top graph in Fig. 1A). Data from RT-PCR analysis using a different PCR primer set (Materials and Methods) showed the same two-peaked pattern (Fig. S1A).

For comparison, we measured mRNA levels of brain-derived neurotrophic factor (Bdnf) in the same set of cortical samples. Unlike Shh mRNA expression, Bdnf mRNA was undetectable before birth at all embryonic ages examined (middle graph in Fig. 1A). But similar to Shh mRNA expression, Bdnf mRNA level increased continuously during postnatal development and reached a peak level between p21 and p30 (middle graph in Fig. 1A). Knowing that synapses develop rapidly during postnatal development (Petralia et al., 1999; Sans et al., 2000), we measured mRNAs of two well-characterized synaptic proteins, bassoon and synaptophysin (Gundelfinger et al., 2016; Kwon and Chapman, 2011). As expected, bassoon and synaptophysin mRNA increased markedly from birth to p21 or p30 (bottom graph in Fig. 1A). Although relative Shh mRNA abundance in the postnatal cortex was lower compared with mRNA for Bdnf and synaptic markers, the temporal expression pattern of Shh mRNA was nearly identical to that of Bdnf and synaptic markers.

We examined two additional brain regions, the hippocampus and cerebellum, concentrating on postnatal ages. Similar to the cortex, Shh mRNA levels in both hippocampus and cerebellum increased steadily during postnatal development (Fig. 1B). At p30, the level of Shh mRNA was significantly higher than the level at birth (p30 versus p1 hippocampus, 4.2±0.7 versus 1, *P*=0.0465; p30 versus p1 cerebellum, 3.9±0.2 versus 1, *P*=0.0069; Fig. 1B).

We subsequently examined Shh protein level in the embryonic and postnatal cortex. We used a commercially available antibody to the N-terminal epitope of Shh (see Materials and Methods). We refer to this antibody as the ShhN antibody. We first evaluated the ShhN antibody by immunoblotting cell lysates from human embryonic kidney (HEK) cells that were transfected with the expression construct either for wild-type full-length Shh protein (ShhFL), or N-terminal fragment of Shh protein (ShhN) (Materials and Methods). As revealed in the blot shown in Fig. 1C, the antibody specifically recognized the expected ∼45 kD ShhFL and ∼19 kD ShhN (Lee et al., 1994). The lack of any Shh protein band from the vector-transfected cells validated the specificity of the ShhN antibody, as HEK cells do not produce endogenous Shh (Chen et al., 2002a,b). In brain tissue extracts, the antibody detected the ∼19 kD ShhN, not the ∼45 kD ShhFL (Fig. 1D; with a similar finding in mouse brain extracts, see Fig. S1B). A different ShhN antibody (5E1; Ericson et al., 1996; Petralia et al., 2011b) (Fig. S1C) similarly showed that the predominant form of Shh protein in brains is the ∼19 kD ShhN. Therefore, we focused on examining this Shh form in brains at different ages.

The temporal expression pattern of ShhN protein mirrored the pattern of Shh mRNA in the cortex: both were highly expressed at e14, gradually declined to a nearly undetectable level at birth, and steadily increased during postnatal development (Fig. 1E; Fig. S1D). Moreover, in postnatal hippocampus and cerebellum, again the protein was low in p1, but by p30, ShhN protein level was significantly higher than in p1 (p30 versus p1 hippocampus, 61±11.7 versus 7±3, *P*=0.037; p30 versus p1 cerebellum, 105.5±17.9 versus 17.5±6.9, *P*=0.0108; Fig. 1F,G). Together, these observations supported significant expression of Shh mRNA and ShhN protein in postnatal and young adult rat brain including the cortex, hippocampus and cerebellum. The closely correlated temporal expression pattern between mRNA and protein suggested that transcription regulation contributed at least in part to the level of Shh protein.

### ShhN protein level in hippocampal neurons

**Fig. 2.**
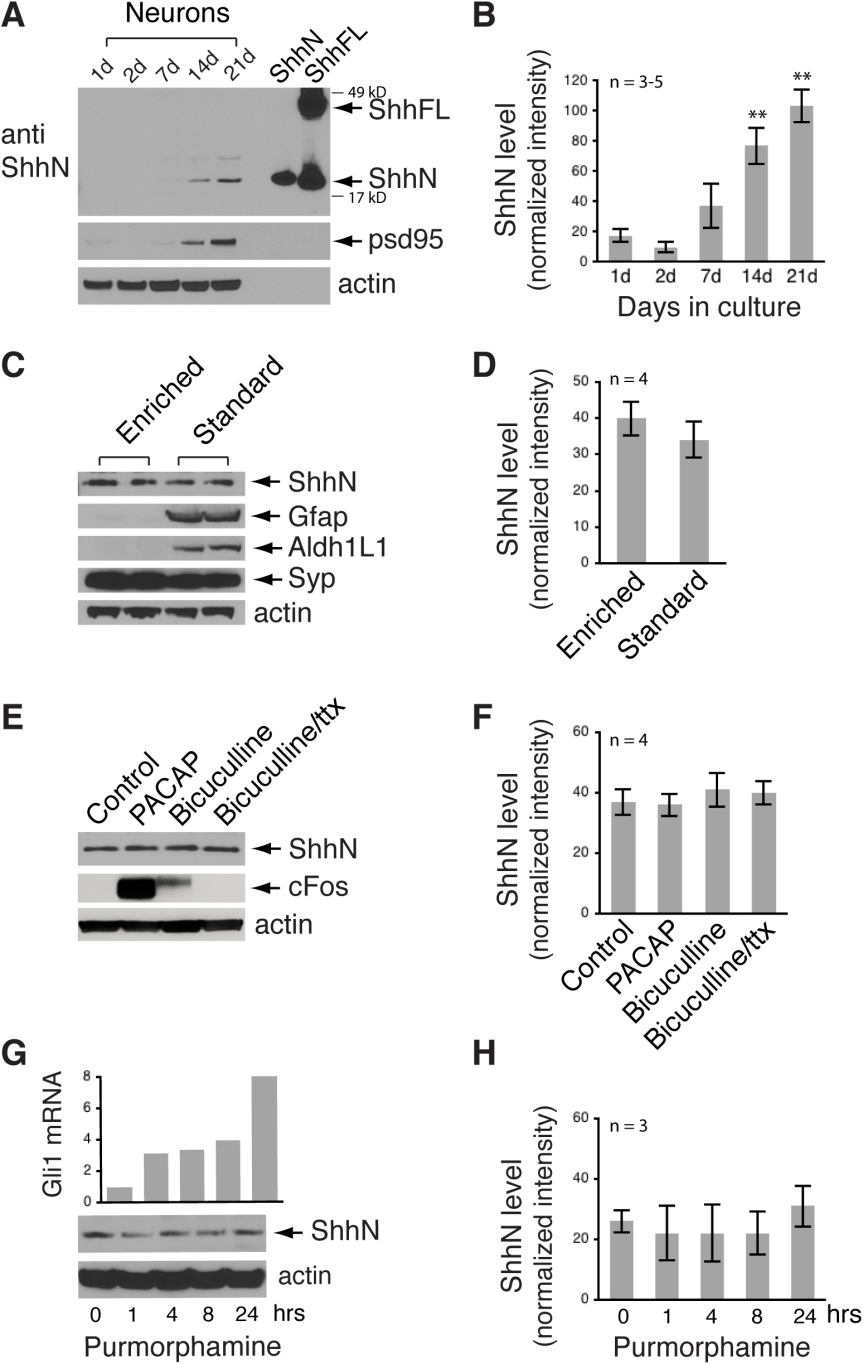
**ShhN protein expression levels in cultured hippocampal neurons.** (A) Immunoblot showing increased ShhN protein level in hippocampal neurons as these neurons matured in culture. d, days in culture. Proteins from HEK cells expressing N-terminal fragment of Shh (ShhN) or full-length Shh (ShhFL) assayed in the same blot as controls. The ShhN antibody detects N-terminal fragment Shh (∼19 kD) as well as full-length Shh (∼45 kD) from HEK cell lysates, whereas in lysates of cultured neurons, ShhN is the dominant Shh species. psd95, a synaptic marker. Additional blots are shown in Fig. S2A,B. (B) Quantification of A. *n*=number of independent cultures. (C) Immunoblot showing similar ShhN protein level in neuron-enriched cultures (Enriched) and standard cultures (Standard). Gfap and Aldh1L1 are glial markers, Syp, synaptophysin, neuronal synaptic marker. (D) Quantification of C. *n*=number of independent cultures. (E,F) Neural activity does not change overall ShhN level. PACAP (10 nM), 2 h; Bicuculline (40 µM), 12 h; ttx, tetrodotoxin (2 µM). (G,H) While treatment of cultured neurons with Shh pathway agonist Purmorphamine (3.6 µM) robustly increases Gli1mRNA level (top, bar graph), the overall level of ShhN protein is not changed (bottom, representative blot) and H. Error bars represent s.e.m. **P<0.01, unpaired *t*-test. The values of ShhN protein level represent the ShhN band intensities normalized to the actin band intensities.

Having found progressively increased expression of endogenous ShhN in the hippocampus during postnatal development, we examined the abundance of this protein in hippocampal neuronal cultures, a model system in which developmental stages of neurons are well described (Dotti et al., 1998; Goslin and Banker, 1989). Immunoblots using the ShhN antibody showed that ShhN was also the most prominent Shh protein in neuronal lysates (Fig. 2A; Fig. S2A), similar to what we observed in brain extracts (Fig. 1; Fig. S1). Therefore, we focused on examining ShhN in these neurons. Comparing with the same amount of total protein (30 µg) from the lysates, ShhN was undetectable in very young neurons (1 or 2 days in culture), low but detectable as neurons grew (7 days in culture), and easily detectable in mature neurons (14 to 21 days in culture) (21 days versus 1 day, 103±11 versus 17.3±4.4; *P*=0.0022; Fig. 2A,B; Fig. S2B).

We wondered whether there were any detectable amounts of extracellular Shh in these hippocampal neurons as they matured in culture. We collected the media from the neurons at various ages and measured ShhN by immunoblots. In spite of concentrating the media (first 25-fold and then tenfold), immunoblots did not show Shh from neurons of any culture ages (Fig. S2C).

Because standard hippocampal neuron cultures comprise not only neurons but also glia, such as astrocytes, we sought to measure ShhN levels in neuron-enriched cultures by treating the standard cultures with AraC (Materials and Methods). Although treatment of AraC eliminated most astrocytes as demonstrated by the disappearance of Gfap and Aldh1L1, markers for astrocytes (Cahoy et al., 2008), ShhN levels were similar between neuron-enriched cultures and the standard cultures (Fig. 2C,D). These results confirmed that neurons produced Shh. Therefore, we used the standard cultures in the remaining experiments of this study.

Given that neural activity upregulates Bdnf mRNA expression (Patterson et al., 1992; Ghosh et al., 1994; Will et al., 2013), we next investigated whether enhanced neural activity alters basal ShhN level in hippocampal neurons. We stimulated the neurons by treating them with bicuculline (a GABA_A_ receptor antagonist) or PACAP (the neuropeptide pituitary adenylate cyclase activating polypeptide) (Baxter et al., 2011; Will et al., 2013). Although cFos level, a sensitive readout for neuronal activity, was markedly increased in the treated neurons, the ShhN level remained unchanged (Fig. 2E,F). Thus, our data suggested that the endogenous global level of ShhN in neurons was not regulated by neural activity.

Finally, we asked if the activity of the Shh pathway itself affected ShhN level in hippocampal neurons. We treated the neurons with Purmorphamine, a Shh agonist (Sinha and Chen, 2006). In the parallel samples, we measured Gli1 mRNA, a sensitive and reliable readout of the Shh pathway activity (Ingham and McMahon, 2001; Varjosalo and Taipale, 2008). RT-PCR analysis showed a time-dependent increase in Gli1 mRNA upon Purmorphamine treatment with an ∼eightfold higher level than untreated control after 24 h (top graph in Fig. 2G). Despite robustly enhanced Shh pathway activity, Purmorphamine treatment did not change the basal ShhN level in these neurons (Fig. 2G,H).

### Shh protein spatial distribution in hippocampal neurons

In *Drosophila* photoreceptor neurons, different fragments of Hedgehog (Hh) protein are segregated in different parts of the cells (Chu et al., 2006; Daniele et al., 2017). We wanted to know if in mammalian neurons, various Shh protein fragments or forms preferentially localize to particular neuronal compartments. For this analysis, we examined the well-defined subcellular compartments in the cultured hippocampal neuron (Dotti et al., 1998; Goslin and Banker, 1989).

**Fig. 3.**
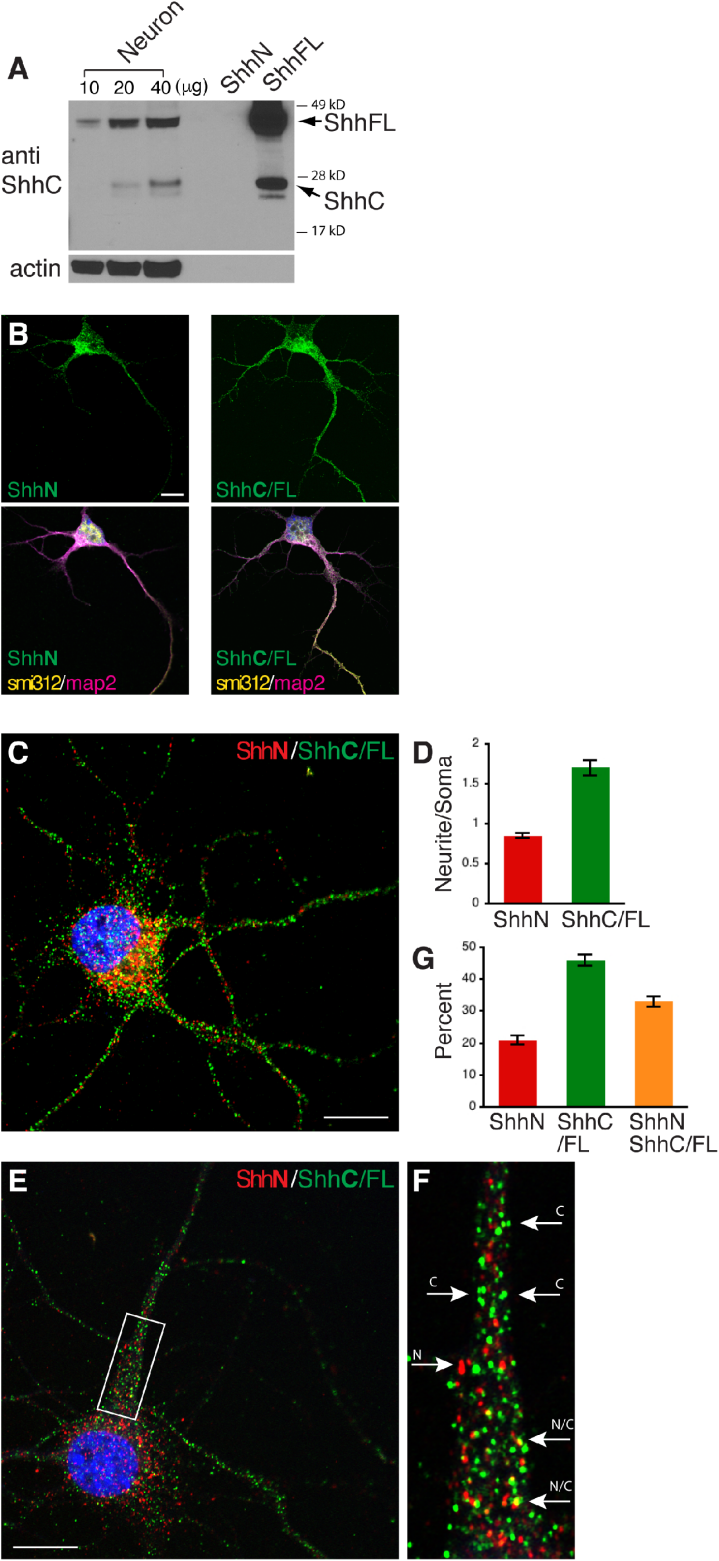
**ShhN and ShhC/FL distribution in cultured hippocampal neurons.** (A) Immunoblot with an antibody to C-terminal epitope of Shh (amino acids 199-437 of mouse Shh). Lanes contain lysates from hippocampal neurons (21 days in culture; 10–40 µg total proteins), and from HEK cells expressing the N-terminal fragment of Shh (ShhN) or full-length Shh (ShhFL). Whereas the ShhC antibody does not detect the N-terminal Shh fragment (∼19 kD) as expected, the antibody detects the full-length Shh (∼45 kD) and C-terminal Shh fragment (∼25 kD) from both ectopically expressed and endogenous Shh. Additional blot of ectopically expressed Shh is shown in Fig. S3A. (B) Fluorescent images of hippocampal neurons co-labeled for ShhN (green) or ShhC/FL (green), an axonal marker smi312 (yellow) and a dendritic marker map2 (magenta). (C) Representative image of a hippocampal neuron showing a trend of higher ShhN (red) immunofluorescence intensity in soma (cell body) but a relatively even ShhC/FL (green) distribution throughout neurites. Additional examples are shown in Fig. S3B. (D) Comparing neurite to soma ratio of ShhN and ShhC/FL fluorescence intensity. *n*=30 cells from three cultures. (E,F) Representative image of a hippocampal neuron with an enlarged area (F) showing examples of ShhN-alone immunolabeled puncta (arrow labeled N), ShhC/FL-alone puncta (arrows labeled C), or ShhN and ShhC/FL puncta next to each other (arrows labeled N/C). (G) Quantification of E and F. *n*=30 cells from three cultures. Scale bars: 10 µm.

In addition to the ShhN antibody which we have characterized (Figs 1C,D, 2A; Figs S1B, 2A), we tested an antibody to a C-terminal epitope of Shh which we refer to as ShhC antibody (see Materials and Methods). Immmunoblots of cell lysates from HEK cells transfected with ShhFL showed that the ShhC antibody detected ShhFL (∼45 kD), and a protein band at ∼25 kD (Fig. 3A; Fig. S3A), the expected size for ShhC protein fragment (Lee et al., 1994). In contrast, and as expected, the ShhC antibody did not detect the ∼19 kD ShhN from HEK cells that were transfected with ShhN (Fig. 3A; Fig. S3A), supporting the specificity of the ShhC antibody in detecting ShhC in addition to ShhFL.

In neuronal lysates, immunoblot with the ShhC antibody revealed that ShhC (∼25 kD) co-migrated with the ShhC from the expressed construct (Fig. 3A). Notably, in immunoblots of both neurons and transfected HEK cells, ShhC levels were lower than the levels of ShhFL and appeared as double bands in some cases. The unambiguous detection of ShhN by the ShhN antibody (Fig. 2A) and ShhC by the ShhC antibody (Fig. 3A) indicate that both of these Shh forms are produced in neurons, as shown for other types of cells (Chang et al., 1994; Lee et al., 1994). Although the ShhN and ShhC must be derived from ShhFL (Lee et al., 1994), no definitive answer about the abundance or presence of neuronal ShhFL can be deduced from our immunoblots because this FL form was detected with the ShhC antibody (Fig. 3A) but not with the ShhN antibody (Fig. 2A).

We next visualized and compared spatial distributions of ShhN and ShhC in hippocampal neurons. Using immunolabeling analysis, we first examined hippocampal neurons that had grown in culture for 2–3 days because neurons at this stage have developed distinctive axons and dendrites (Dotti et al., 1998; Goslin and Banker, 1989). We co-immunolabeled the neurons with the antibody for ShhN or ShhC, and with antibodies to an axonal marker smi312 and dendritic marker map2 (Fig. 3B). Because immunoblots of neuronal lysates showed the ShhC antibody detecting both ShhC and ShhFL (Fig. 3A), ShhC immunolabeling signals could be indicative of ShhC or ShhFL or both; therefore, we designated ShhC-immunolabeled signals as ShhC/FL.

Overall, neither ShhN nor ShhC/FL immunolabeling displayed any noticeable spatial distribution differences between axons, dendrites or soma. Upon closer inspection, however, we noticed that ShhN immunolabeling tended to be higher in soma and proximal regions of axons and dendrites, whereas ShhC/FL immunolabeling was more uniform and widespread throughout the entire neurons (Fig. 3B).

When we co-labeled for ShhN and ShhC/FL in slightly older neurons (7 days in culture), the difference in the overall distribution pattern between the two Shh forms became more apparent: ShhN appeared higher in the soma than in neurites, whereas ShhC/FL labeling intensity was uniform between the soma and neurites in some cells or higher in neurites than in the soma in other cells (Fig. 3C; Fig. S3B). Quantitative assessment of immunofluorescence intensity expressed as neurite to soma ratio confirmed our visual impression: ShhN was higher in soma than neurites whereas ShhC/FL on average was higher in neurites than soma (Fig. 3D).

Further examination of individual ShhN or ShhC/FL immunolabeled dots or puncta revealed three populations: ShhN alone, ShhC alone, and ShhN and ShhC/FL co-localized or closely opposed to each other (Fig. 3E,F). Counting the puncta number in the proximal area of neurites (i.e. white box in Fig. 3E) showed ∼20% ShhN puncta, ∼45% ShhC/FL puncta, and ∼35% ShhN and ShhC/FL co-labeled puncta (Fig. 3G). The overlapping ShhN and ShhC/FL fluorescent signals imply a physical association between the different Shh forms. The dually labeled puncta may represent various combinations of ShhFL, ShhN and ShhC.

### Shh protein distribution in synapses of hippocampal neurons

**Fig. 4.**
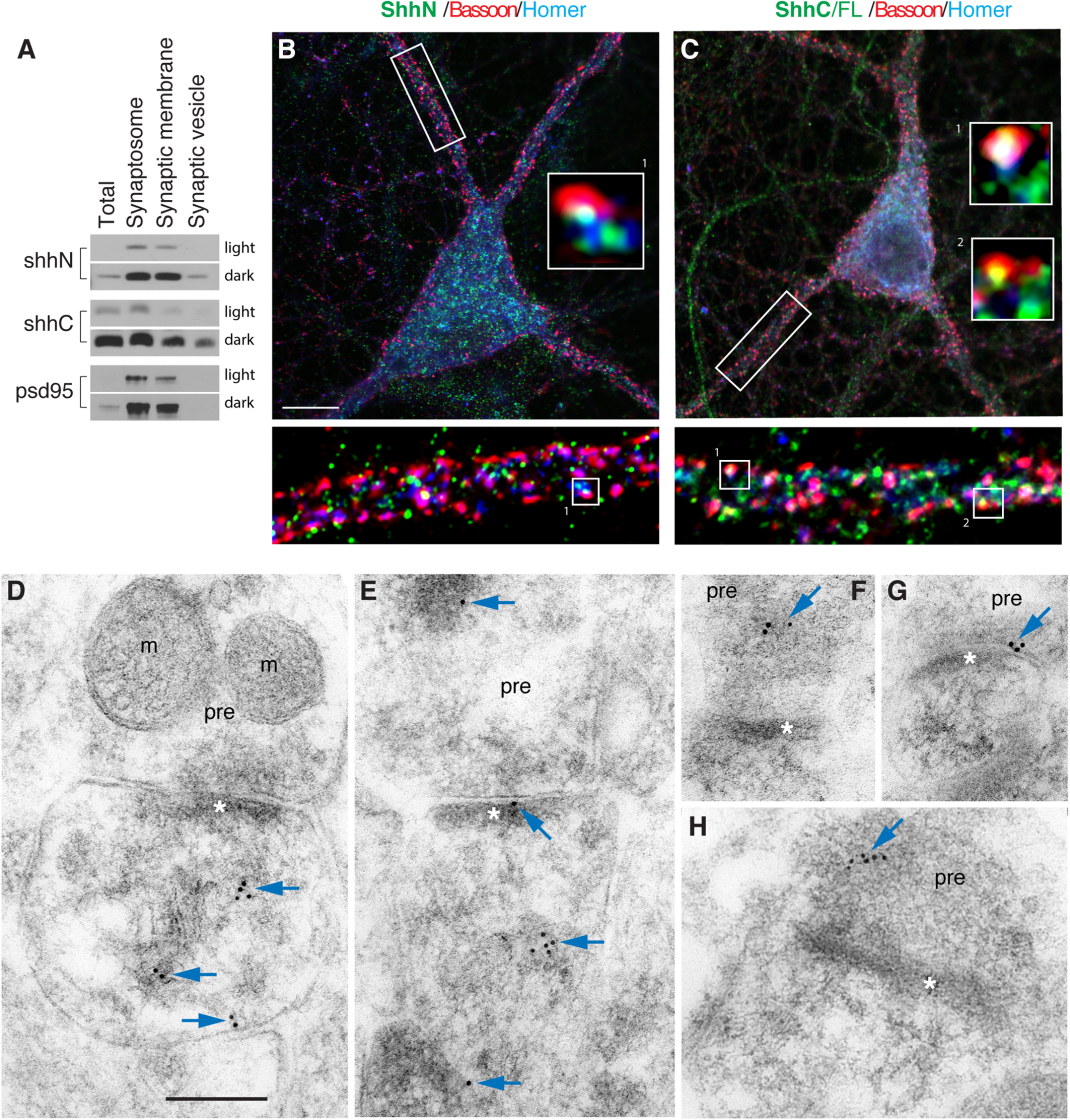
**ShhN and ShhC localization in synapses of hippocampal neurons.** (A) Immunoblots of synaptic preparations from rat brains showing the presence of ShhN and ShhC in synapses. psd95, a synaptic marker. Blots of two different exposures are shown. (B,C) Fluorescent images of mature hippocampal neuron (21 days in culture) co-labeled for ShhN (green in B) or ShhC/FL (green in C), a presynaptic marker, bassoon (red), and a postsynaptic marker, homer (blue). Box1 in B shows a zoomed-in view of ShhN immunolabeling in close proximity to bassoon and homer. Box1 and 2 in C are zoomed-in views of ShhC/FL immunolabeling also in close proximity to bassoon and homer. Scale bar: B,C: 10 µm. Additional examples are shown in Fig. S4A,B. (D–H) Immunogold localization of ShhC/FL in the CA1 stratum radiatum of the mouse hippocampus, with 10 nm immunogold (arrows). Labeling is found both in the postsynaptic spine (D,E) and presynaptic terminal (pre; E–H). Note localizations on postsynaptic tubulovesicular structures in D and E, and associated with the postsynaptic (E), or presynaptic (G) membrane; in the latter case, it is just perisynaptic to the active zone. m, mitochondrion; asterisk, postsynaptic density. Scale bar: 200 nm. Additional examples are shown in Fig. S4C–I.

Based on the observation that ShhN and ShhC were detected in synaptosomal preparations of rat brains (Fig. 4A; ShhFL is not shown), we visualized the distribution of ShhN and ShhC/FL in synapses of mature hippocampal neurons (at least 21 days in culture). We co-immunolabeled ShhN or ShhC/FL with a presynaptic marker, bassoon and a postsynaptic marker, homer (Fig. 4B; Fig. S4A). Using confocal microscopy with Airyscan (Zeiss 880, with ∼140 nm xy and 400 nm z resolution), we observed that ShhN and ShhC/FL immunolabeling were similarly juxtaposed to bassoon and homer (Fig. 4B; Fig. S4A). Although our observations indicate that both forms of Shh are closely associated with synapses, it is important to note that confocal microscopy even with Airyscan does not offer sufficient resolution to definitively resolve the compartments of single synapses.

In order to precisely reveal individual synapses, we next performed immunogold electron microscopy (immunoEM) of hippocampal neurons in brain tissues. Our previous immunoEM study of hippocampal neurons in mature brains showed that ShhN localized to both pre- and postsynaptic compartments (Petralia et al., 2011b). Here, we focused on analyzing ShhC/FL. Similar to our previous findings of ShhN (Petralia et al., 2011b), ShhC/FL immunogold labeling was seen in the presynaptic terminals and postsynaptic spines of hippocampal neurons in mature brains (Fig. 4D–H; Fig. S4C–I). Therefore, in terms of synaptic distribution of Shh proteins, ShhN and ShhC/FL localize on both sides of the synapse.

### Effects of fasting and exercise on Shh levels in mouse hippocampus

**Fig. 5.**
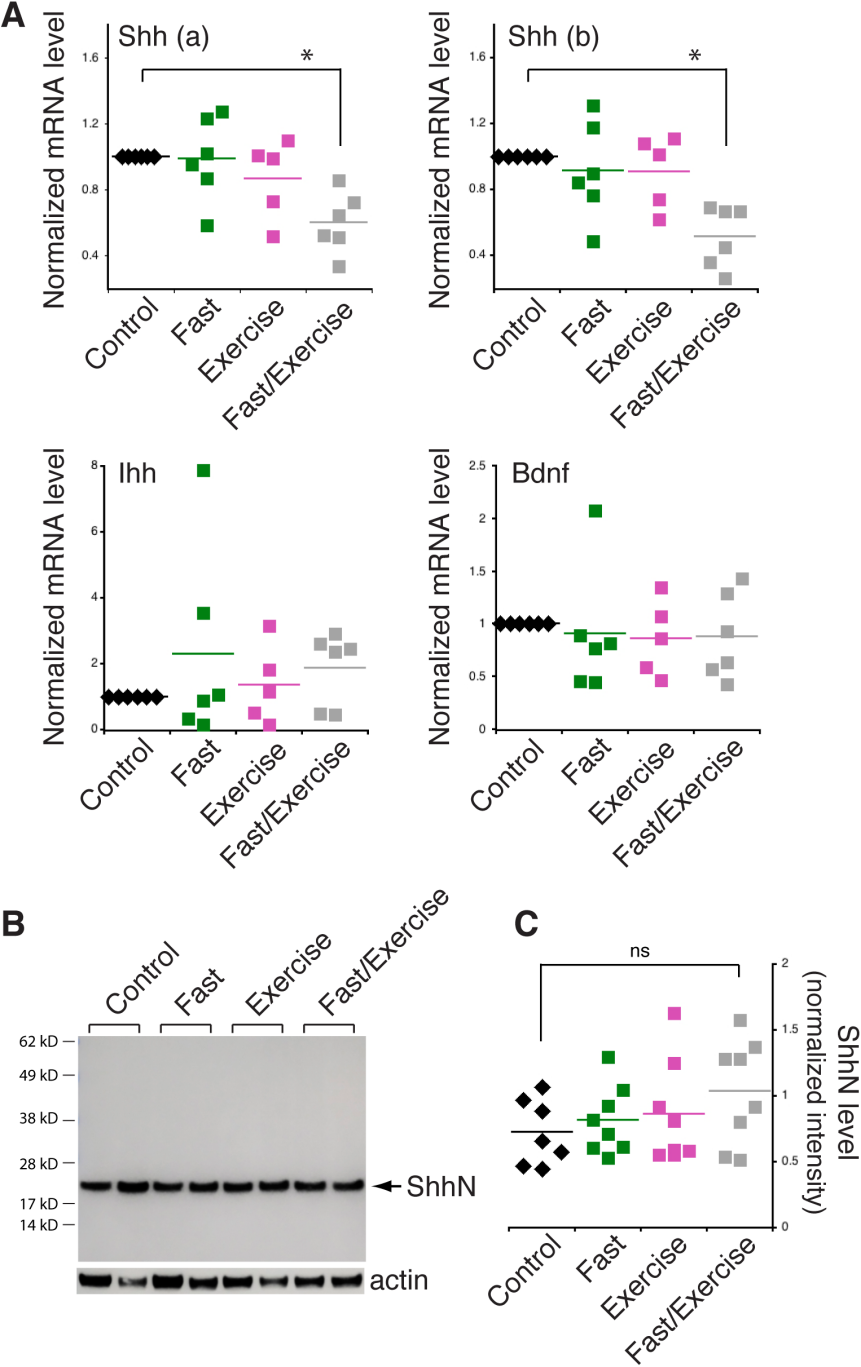
**Shh mRNA and ShhN protein levels in mouse hippocampus after fasting or exercising.** (A) Quantitative RT-PCR assay of Shh mRNA level in hippocampal tissues from mice subjected to fasting, exercise or fasting plus exercise. Top two graphs were the same hippocampal mRNA samples but amplified by using different PCR primers to Shh. The same hippocampal samples were also analyzed for Ihh (Indian hedgehog) and Bdnf mRNA. Each symbol represents an individual mouse. *P<0.05, unpaired *t*-test. (B) Representative immunoblot showing similar levels of ShhN protein in the hippocampal tissues of mice between different conditions. C is quantification of B. Each symbol represents an individual mouse. ns, not statistically significant.

We went on to test whether fasting or exercise affected Shh mRNA level in mouse hippocampus. After subjecting mice to fasting or exercise (Marosi et al., 2018; also see Materials and Methods), we dissected the hippocampi and extracted mRNA or proteins for analysis. Using quantitative RT-PCR to analyze Shh mRNA, we found no significant changes in the hippocampi of mice subjected to fasting or exercise compared to control mice (Fig. 5A). When mice were subjected to fasting plus exercise, however, Shh mRNA level in the hippocampus was significantly reduced (Fig. 5A, top left). Consistently, RT-PCR analysis of the samples using a different PCR primer set produced the same results (Fig. 5A, top right). In contrast, mRNA level of Indian hedgehog (Ihh), another member of hedgehog family, was not different between mouse groups (Fig. 5A, bottom left). Likewise, Bdnf mRNA also was unchanged under any of the conditions (Fig. 5A, bottom left).

Finally, we examined Shh protein levels in the hippocampus of these mice. We decided to focus on examining ShhN protein because the ShhN antibody revealed ShhN protein specifically and performed reliably when analyzing mouse brain samples by immunoblotting (Fig. 5B). We found that, unlike Shh mRNA, ShhN protein level in the hippocampal samples was not appreciably altered in any of the mouse groups (eight mice for each group, Fig. 5C). The lack of a correlation between global Shh mRNA and protein can be caused by several factors such as protein turnover and other post-transcription mechanisms. Further experiments are required to address these questions.

## DISCUSSION

We have provided evidence that postnatal neurons, including mature hippocampal neurons, produce an appreciable amount of Shh protein. When we systematically evaluated the abundance of Shh in brains of multiple ages from embryonic day 14 to postnatal day 30, we identified an embryonic age-dependent decline followed by a postnatal age-dependent resurgence. The postnatal, progressively-increased Shh expression coincides with brain development during which both neurons and glia are growing rapidly. The lack of change in Shh levels in cultured neurons after removing glia (Fig. 2C,D) suggests that neurons produce Shh. It will be important to determine that, at least in the hippocampus, if neurons are the sole Shh producers, and if all different subpopulations of neurons contribute to Shh production.

Among many questions that remain to be answered is whether hippocampal Shh signaling is autonomous or nonautonomous, or both. Hippocampal neurons possess the Shh pathway receptor, Patched and transducer, Smoothened, and preferentially traffic them to the dendrites or dendritic spines (Petralia et al., 2011a), coincidentally the classical signal receiving sites for some neurotransmitters. In response to exogenously applied Shh, hippocampal neurons extend axons and grow more mature presynapses with a concomitant increase of Gli1 (Mitchell et al., 2012; Yao et al., 2015). It had been unclear, however, where in the hippocampus the endogenous Shh came from. Our current finding of Shh being produced by hippocampal neurons themselves points to a model in which Shh, either from neighboring or the same neurons, and alone or together, acts on recipient neurons and carries out functions accordingly. Another related and important future question is whether in neurons all Shh protein forms are present and functional. The abundance of ShhN in neurons suggests that this form is likely a functionally active form, as it is known for other types of cells (Chang et al., 1994; Lee et al., 1994). Given the implications that ShhFL has functions in some cells (Tokhunts et al., 2010), and ShhC may be the extracellularly-released form (Lee et al., 1994; Bumcrot et al., 1995), it will be interesting and necessary to pinpoint in the future the exact Shh biogenesis process in hippocampal neurons, and determine if the distinct Shh forms have specialized functions.

## MATERIALS AND METHODS

### Animals

All animal procedures were approved by the NIA Animal Care and Use Committee and complied with the NIH Guide for Care and Use of Laboratory Animals. Timed pregnant female Sprague-Dawley rats were used as the source of embryonic brain tissues. Postnatal rats (postnatal day 1 to p30) of either sex were used as the source of cortical, cerebellar and hippocampal tissues. Hippocampi of 8-week-old male C57BL/6 mice from a previous study (Sun et al., 2011) were used for immunogold labeling. Hippocampi of 6-month-old male C57BL/6 mice also from a previous study (Marosi et al., 2018) were used for fasting and exercise experiments.

### Reagents

Anti-ShhN (#2207; epitope surrounding Glu53 of human Shh) and cFos (#2250S) were from Cell Signaling Technology. Anti-ShhN (5E1) was from the Developmental Studies Hybridoma Bank. Anti-ShhC (#S9947; epitope-amino acids 199-437 of mouse Shh), anti-synaptophysin (#S5768), anti-map2 (#M9942) and anti-actin (#A5441 and #A2066) were from Sigma-Aldrich. smi312 antibody (#SMI-312R) was from Convance/BioLegend. Anti-bassoon (#141002), anti-homer1 (#160004), anti-Aldh1L1 (#278003), and anti-Gfap (#173002) were from Synaptic Systems. Anti-psd95 (#7E3-1B8) was from Thermo Fisher Scientific. Bicuculline (#0130) and PACAP (#1183) were from Tocris Bioscience. Purmorphamine (#ALX-420-045) was from Enzo Biochem Inc. Cytosine β-D-arabinofuranoside (#C1768) and Tetrodotoxin (#T5551) were from Sigma-Aldrich.

### Hippocampal neuron culture

Cultures of hippocampal neurons were prepared from embryonic day 18 rat hippocampi as described (Kaech and Banker, 2006; Yao et al., 2015). Dissociated neurons were seeded at low density (∼50 cells mm^−2^) for immunofluorescence, and high density (150–200 cells mm^−2^) for immunoblot and RT-PCR analysis. The neurons were grown in neurobasal medium supplemented with B27 (Invitrogen). For immunofluorescence, the neurons were grown on polylysine (1 mg ml^−1^)-coated glass coverslips (no 1.5). For immunoblotting and RT-PCR, the neurons were grown in polylysine-coated plastic dishes (0.1 mg ml^−1^). Neuron-enriched cultures were prepared following a protocol (Tushev et al., 2018) with minor modifications. Briefly, one day after initial cell seeding, 2.5 µM of cytosine β-D-arabinofuranoside (AraC) was added. Forty-eight hours later, araC-containing medium was replaced by the growth medium composed of 50% fresh neurobasal medium with B27, 25% of conditioned medium from cortical neurons, and 25% of conditioned medium from glial cells.

### DNA constructs and transfection

The full-length and N-terminal fragment of mouse Shh constructs were kindly provided by Dr James K. Chen (Stanford University). Human embryonic kidney (HEK) 293 cells obtained from ATCC were cultured according to the manufacturer's instructions and transfected using a calcium phosphate-based kit (CalPhos mammalian transfection kit; #631312, Clontech).

### Quantitative PCR (RT-qPCR)

Total RNA was extracted from brain extracts or cell pellets of cultured neurons with RNeasy Mini Kit (Qiagen). RNA concentration was measured with NanoDrop 2000. Five hundred nanograms of total RNA was reverse transcribed using SuperScript III First-Strand Synthesis System (Invitrogen). qPCR was carried out using SYBR Green master mix on 3 µl of cDNA, and analyzed with ViiA 7 (Applied Biosystems). Primer sequences used were as follows: *Shh (d)*: 5′-TCAGAGGTGCAAAGACAAGTTA-3′ and 5′-ACCCTCATAGTGTAGAGACTCC-3′; *Shh (e)*: 5′-GCCGATATGAAGGGAAGATCAC-3′ and 5′-GGAGATGGCCAAGGCATTTA-3′; *Gli1*: 5′-TCGACCTGCAAACCGTAATC-3′ and 5′-CATCTGAGGTGGGAATCCTAAAG-3′; *bassoon*: 5′-CAGCTACGAGCACGGTAAAG −3′ and 5′-TGGGAGTCAGAGGGATATGTAG-3′. The following primers were from QuantiTech primer assays (Qiagen): *Shh (f)*, # QT00184912; *Bdnf*: # QT00375998; *synaptophysin*: # QT02338056. Primer sequences for *Ihh* were described in Lu et al. (2018). ΔΔCt analysis was used to normalize target gene expression to RPLO reference gene expression. Target gene expression of embryonic and postnatal brain tissues was then normalized to expression at postnatal day 1 (p1).

### Immunoblot analysis

Tissues or cell pellets were sonicated in RIPA buffer (#89900, Thermo Fisher Scientific) containing protease and phosphatase inhibitors (#78444, Thermo Fisher Scientific). Following centrifugation at 10,000 ***g*** for 10 min at 4°C, the supernatant was collected and the amount of total proteins was estimated with a Pierce BCA protein assay kit (Pierce Biotechnology). Protein samples were separated by 4–20% Bis-Tris SDS-PAGE and transferred to nitrocellulose membranes. Following incubation with blocking buffer (5% dry milk and 0.05% Tween20 in PBS), the membranes were incubated overnight at 4°C in the blocking buffer containing one of the following antibodies: Shh 5E1 at 1:250; ShhC and Aldh1L1 at 1:500; ShhN, psd95, Gfap, Aldh1L1, and cFos at 1:1000; synaptophysin and actin at 1:5000. The membranes were then washed (0.1% Tween20 in PBS) and incubated with appropriate peroxidase-conjugated secondary antibodies. The proteins were visualized using a chemiluminescence kit from Pierce or Kindle Biosciences. The intensity of protein bands was analyzed using ImageJ software.

Synaptosomal preparations were purchased from Synaptic Systems. Twenty micrograms of protein from each sample were used in immunoblots. For immunoblots of extracellular ShhN, the culture media were concentrated 25-fold and then tenfold using Amicon centrifugal filters (10 kD). Thirty micrograms of protein from each sample were analyzed by immunoblots.

### Immunocytochemistry, fluorescence microscopy and image analysis

Immunofluorescence labeling was performed as previously described (Yao et al., 2015). Briefly, neurons were fixed in 4% paraformaldehyde and 4% sucrose for 15 min. Following permeabilization in 0.2% Triton X-100 and blocking in 10% BSA, the neurons were incubated with antibody to ShhN (1:250), ShhC (1:250), smi-312 (1:1000), map2 (1:2000), bassoon (1:250), or homer (1:250). After washing, the neurons were incubated with appropriate fluorescence-tagged secondary antibodies. The glass coverslips containing the labeled neurons were mounted in Prolong Gold antifade reagent (Thermo Fisher Scientific).

Confocal images were acquired with an Apochromat 63×/1.4 numerical aperture objective lens on a Zeiss LSM 880 microscope with Airyscan (Carl Zeiss). For each antibody labeling, the image acquisition settings were kept the same between different experiments. The brightness and contrast levels of the images were adjusted in Adobe Photoshop, and compiled in Adobe Illustrator. No additional digital image processing was performed. Control cells omitting the primary antibody showed no fluorescence labeling.

We used ImageJ (NIH) software to compare the immunofluorescence intensity for ShhN or ShhC in soma and neurites. We also estimated the relative proportion of immunolabeled puncta for ShhN, ShhC or co-localized ShhN and ShhC (touching or overlapping green and red pixels). Randomly selected neurons from three different cultures were used for the analysis. For each neuron, 100 immunolabeled puncta were counted. Counting was performed by an observer (E.C.) who was familiar with the analyzing criteria, but who was unaware of the experimental details until after analyses were complete.

### Immunoelectron microscopy

Post-embedding immunogold labeling was performed as described previously (Petralia et al., 2010; Yao et al., 2015). Following perfusion with 4% PFA plus 0.5% glutaraldehyde, mouse brain tissue was cryoprotected and frozen in a Leica CPC and then processed for freeze-substitution in Lowicryl HM-20 resin in a Leica AFS. Thin sections were incubated in 10% normal goat serum, then overnight in primary antibody, and followed by incubation for 1 h with 10 nm immunogold. Sections were stained with uranyl acetate and lead citrate. Control immunogold labeling of sections omitting the primary antibody showed only rare gold particles.

### Presentation of data and statistics

All graphs were produced using KaleidaGraph (Synergy) software. Statistical comparisons were calculated using the unpaired Student's *t*-test. All results are expressed as mean±s.e.m.

## Supplementary Material

Supplementary information

## References

[BIO040592C1] Baxter P. S., Martel M.-A., McMahon A., Kin P. C. and Hardingham G. E. (2011). Pituitary adenylate cyclase-activating peptide induces long-lasting neuroprotection through the induction of activity-dependent signaling via the cyclic AMP response element-binding protein-regulated transcription co-activator 1. *J. Neurochem.* 118, 365-378. 10.1111/j.1471-4159.2011.07330.x 21623792 PMC3557719

[BIO040592C2] Bumcrot D. A., Takada R. and McMahon A. P. (1995). Proteolytic processing yields two secreted forms of sonic hedgehog. *Mol. Cell Biol.* 15, 2294-2303. 10.1128/MCB.15.4.2294 7891723 PMC230457

[BIO040592C3] Cahoy J. D., Emery B., Kaushal A., Foo L. C., Zamanian J. L., Christopherson K. S., Xing Y., Lubischer J. L., Krieg P. A., Krupenko S. A. et al. (2008). A transcriptome database for astrocytes, neurons, and oligodendrocytes: a new resource for understanding brain development and function. *J. Neurosci.* 28, 264-278. 10.1523/JNEUROSCI.4178-07.2008 18171944 PMC6671143

[BIO040592C4] Chang D. T., López A., von Kessler D. P., Chiang C., Simandl B. K., Zhao R., Seldin M. F., Fallon J. F. and Beachy P. A. (1994). Products, genetic linkage and limb patterning activity of a murine hedgehog gene. *Development* 120, 3339-3353. 7720571 10.1242/dev.120.11.3339

[BIO040592C5] Charron F., Stein E., Jeong J., McMahon A. P. and Tessier-Lavigne M. (2003). The morphogen sonic hedgehog is an axonal chemoattractant that collaborates with netrin-1 in midline axon guidance. *Cell* 113, 11-23. 10.1016/S0092-8674(03)00199-5 12679031

[BIO040592C6] Chen J. K., Taipale J., Cooper M. K. and Beachy P. A. (2002a). Inhibition of Hedgehog signaling by direct binding of cyclopamine to Smoothened. *Genes Dev.* 16, 2743-2748. 10.1101/gad.1025302 12414725 PMC187469

[BIO040592C7] Chen J. K., Taipale J., Young K. E., Maiti T. and Beachy P. A. (2002b). Small molecule modulation of Smoothened activity. *Proc. Natl. Acad. Sci. USA* 99, 14071-14076. 10.1073/pnas.182542899 12391318 PMC137838

[BIO040592C8] Chiang C., Litingtung Y., Lee E., Young K. E., Corden J. L., Westphal H. and Beachy P. A. (1996). Cyclopia and defective axial patterning in mice lacking Sonic hedgehog gene function. *Nature* 383, 407-413. 10.1038/383407a0 8837770

[BIO040592C9] Chu T., Chiu M., Zhang E. and Kunes S. (2006). C-terminal motif targets Hedgehog to axons, coordinating assembly of the Drosophila eye and brain. *Dev. Cell* 10, 635-646. 10.1016/j.devcel.2006.03.003 16678778

[BIO040592C10] Daniele J. R., Chu T. and Kunes S. (2017). A novel proteolytic event controls Hedgehog intracellular sorting and distribution to receptive fields. *Biol. Open* 6, 540-550. 10.1242/bio.024083 28298318 PMC5450321

[BIO040592C11] Dessaud E., McMahon A. P. and Briscoe J. (2008). Pattern formation in the vertebrate neural tube: a sonic hedgehog morphogen-regulated transcriptional network. *Development* 135, 2489-2503. 10.1242/dev.009324 18621990

[BIO040592C12] Dotti C. G., Sullivan C. A. and Banker G. A. (1998). The establishment of polarity by hippocampal neurons in culture. *J. Neurosci.* 8, 1454-1468. 10.1523/JNEUROSCI.08-04-01454.1988 PMC6569279 3282038

[BIO040592C13] Ericson J., Morton S., Kawakami A., Roelink H. and Jessell T. M. (1996). Two critical periods of Sonic Hedgehog signaling required for the specification of motor neuron identity. *Cell* 87, 661-673. 10.1016/S0092-8674(00)81386-0 8929535

[BIO040592C14] Farmer W. T., Abrahamsson T., Chierzi S., Lui C., Zaelzer C., Jones E. V., Bally B. P., Chen G. G., Théroux J.-F., Peng J. et al. (2016). Neurons diversify astrocytes in the adult brain through sonic hedgehog signaling. *Science* 351, 849-854. 10.1126/science.aab3103 26912893

[BIO040592C15] Garcia A. D. R., Petrova R., Eng L. and Joyner A. L. (2010). Sonic hedgehog regulates discrete populations of astrocytes in the adult mouse forebrain. *J. Neurosci.* 30, 13597-13608. 10.1523/JNEUROSCI.0830-10.2010 20943901 PMC2966838

[BIO040592C16] Ghosh A., Carnahan J. and Greenberg M. E. (1994). Requirement for BDNF in activity-dependent survival of cortical neurons. *Science* 263, 1618-1623. 10.1126/science.7907431 7907431

[BIO040592C17] Goslin K. and Banker G. (1989). Experimental observations on the development of polarity by hippocampal neurons in culture. *J. Cell Biol.* 108, 1507-1516. 2925793 10.1083/jcb.108.4.1507 PMC2115496

[BIO040592C18] Gundelfinger E. D., Reissner C. and Garner C. C. (2016). Role of Bassoon and piccolo in assembly and molecular organization of the active zone. *Front. Synaptic Neurosci.* 7, 19 10.3389/fnsyn.2015.00019 26793095 PMC4709825

[BIO040592C19] Hammond R., Blaess S. and Abeliovich A. (2009). Sonic hedgehog is a chemoattractant for midbrain dopaminergic axons. *PLoS ONE* 4, e7007 10.1371/journal.pone.0007007 19774071 PMC2742719

[BIO040592C20] Han Y.-G., Spassky N., Romaguera-Ros M., Garcia-Verdugo J.-M., Aguilar A., Schneider-Maunoury S. and Alvarez-Buylla A. (2008). Hedgehog signaling and primary cilia are required for the formation of adult neural stem cells. *Nat. Neurosci.* 11, 277-284. 10.1038/nn2059 18297065

[BIO040592C21] Ingham P. W. and McMahon A. P. (2001). Hedgehog signaling in animal development: paradigms and principles. *Genes Dev.* 15, 3059-3087. 10.1101/gad.938601 11731473

[BIO040592C22] Kaech S. and Banker G. (2006). Culturing hippocampal neurons. *Nat. Protoc.* 1, 2406-2415. 10.1038/nprot.2006.356 17406484

[BIO040592C23] Kwon S. E. and Chapman E. R. (2011). Synaptophysin regulates the kinetics of synaptic vesicle endocytosis in central neurons. *Neuron* 70, 845-854. 10.1016/j.neuron.2011.04.001 PMC3136197 21658579

[BIO040592C24] Lai K., Kaspar B. K., Gage F. H. and Schaffer D. V. (2003). Sonic hedgehog regulates adult neural progenitor proliferation in vitro and in vivo. *Nat. Neurosci.* 6, 21-27. 10.1038/nn983 12469128

[BIO040592C25] Lee J. J., Ekker S. C., von Kessler D. P., Porter J. A., Sun B. I. and Beachy P. A. (1994). Autoproteolysis in hedgehog protein biogenesis. *Science* 266, 1528-1537. 10.1126/science.7985023 7985023

[BIO040592C26] Lu W.-J., Mann R. K., Nguyen A., Bi T., Silverstein M., Tang J. Y., Chen X. and Beachy P. A. (2018). Neuronal delivery of Hedgehog directs spatial patterning of taste organ regeneration. *Proc. Natl. Acad. Sci. USA* 115, E200-E209. 10.1073/pnas.1719109115 29279401 PMC5777079

[BIO040592C27] Marosi K., Moehl K., Navas-Enamorado I., Mitchell S. J., Zhang Y., Lehrmann E., Aon M. A., Cortassa S., Becker K. G. and Mattson M. P. (2018). Metabolic and molecular framework for the enhancement of endurance by intermittent food deprivation. *FASEB J.* 32, 3844-3858. 10.1096/fj.201701378RR 29485903 PMC5998977

[BIO040592C28] Mitchell N., Petralia R. S., Currier D. G., Wang Y.-X., Kim A., Mattson M. P. and Yao P. J. (2012). Sonic hedgehog regulates presynaptic terminal size, ultrastructure and function in hippocampal neurons. *J. Cell. Sci.* 125, 4207-4213. 10.1242/jcs.105080 22641692 PMC3516435

[BIO040592C29] Parra L. M. and Zou Y. (2010). Sonic hedgehog induces response of commissural axons to Semaphorin repulsion during midline crossing. *Nat. Neurosci.* 13, 29-35. 10.1038/nn.2457 19946319

[BIO040592C30] Palma V., Lim D. A., Dahmane N., Sánchez P., Brionne T. C., Herzberg C. D., Gitton Y., Carleton A., Alvarez-Buylla A. and Ruiz i Altaba A. (2005). Sonic hedgehog controls stem cell behavior in the postnatal and adult brain. *Development* 132, 335-344. 10.1242/dev.01567 15604099 PMC1431583

[BIO040592C31] Patterson S. L., Grover L. M., Schwartzkroin P. A. and Bothwell M. (1992). Neurotrophin expression in rat hippocampal slices: a stimulus paradigm inducing LTP in CA1 evokes increases in BDNF and NT-3 mRNAs. *Neuron* 9, 1081-1088. 10.1016/0896-6273(92)90067-N 1463608

[BIO040592C32] Petralia R. S., Esteban J. A., Wang Y. X., Partridge J. G., Zhao H. M., Wenthold R. J. and Malinow R. (1999). Selective acquisition of AMPA receptors over postnatal development suggests a molecular basis for silent synapses. *Nat. Neurosci.* 2, 31-36. 10.1038/4532 10195177

[BIO040592C33] Petralia R. S., Wang Y. X., Hua F., Yi Z., Zhou A., Ge L., Stephenson F. A. and Wenthold R. J. (2010). Organization of NMDA receptors at extrasynaptic locations. *Neuroscience* 167, 68-87. 10.1016/j.neuroscience.2010.01.022 20096331 PMC2840201

[BIO040592C34] Petralia R. S., Schwartz C. M., Wang Y.-X., Mattson M. P. and Yao P. J. (2011a). Subcellular localization of Patched and Smoothened, the receptors for Sonic hedgehog signaling, in the hippocampal neuron. *J. Comp. Neurol.* 519, 3684-3699. 10.1002/cne.22681 21618238 PMC3196849

[BIO040592C35] Petralia R. S., Wang Y. X., Mattson M. P. and Yao P. J. (2011b). Sonic hedgehog distribution within mature hippocampal neurons. *Commun. Integr. Biol.* 4, 775-777. 10.4161/cib.17832 22446553 PMC3306357

[BIO040592C36] Petralia R. S., Schwartz C. M., Wang Y. X., Kawamoto E. M., Mattson M. P. and Yao P. J. (2013). Sonic hedgehog promotes autophagy in hippocampal neurons. *Biol. Open* 2, 499-504. 10.1242/bio.20134275 23789099 PMC3654269

[BIO040592C37] Sans N., Petralia R. S., Wang Y.-X., Blahos J. II, Hell J. W. and Wenthold R. J. (2000). A developmental change in NMDA receptor-associated proteins at hippocampal synapses. *J. Neurosci.* 20, 1260-1271. 10.1523/JNEUROSCI.20-03-01260.2000 10648730 PMC6774158

[BIO040592C38] Sinha S. and Chen J. K. (2006). Purmorphamine activates the Hedgehog pathway by targeting Smoothened. *Nat. Chem. Biol.* 2, 29-30. 10.1038/nchembio753 16408088

[BIO040592C39] Sun W., Maffie J. K., Lin L., Petralia R. S., Rudy B. and Hoffman D. A. (2011). DPP6 establishes the A-type K(+) current gradient critical for the regulation of dendritic excitability in CA1 hippocampal neurons. *Neuron* 71, 1102-1115. 10.1016/j.neuron.2011.08.008 21943606 PMC3184237

[BIO040592C40] Tokhunts R., Singh S., Chu T., D'Angelo G., Baubet V., Goetz J. A., Huang Z., Yuan Z., Ascano M., Zavros Y. et al. (2010). The full-length unprocessed hedgehog protein is an active signaling molecule. *J. Biol. Chem.* 285, 2562-2568. 10.1074/jbc.M109.078626 19920144 PMC2807313

[BIO040592C41] Tushev G., Glock C., Heumüller M., Biever A., Jovanovic M. and Schuman E. M. (2018). Alternative 3′ UTRs modify the localization, regulatory potential, stability, and plasticity of mRNAs in neuronal compartments. *Neuron* 98, 495-511.e6. 10.1016/j.neuron.2018.03.030 29656876

[BIO040592C42] Varjosalo M. and Taipale J. (2008). Hedgehog: functions and mechanisms. *Genes Dev.* 22, 2454-2472. 10.1101/gad.1693608 18794343

[BIO040592C43] Wechsler-Reya R. J. and Scott M. P. (1999). Control of neuronal precursor proliferation in the cerebellum by Sonic Hedgehog. *Neuron* 22, 103-114. 10.1016/S0896-6273(00)80682-0 10027293

[BIO040592C44] Will T. J., Tushev G., Kochen L., Nassim-Assir B., Cajigas I. J., Tom Dieck S. and Schuman E. M. (2013). Deep sequencing and high-resolution imaging reveal compartment-specific localization of Bdnf mRNA in hippocampal neurons. *Sci. Signal.* 6, rs16 10.1126/scisignal.2004520 24345682 PMC5321484

[BIO040592C45] Yao P. J., Petralia R. S., Ott C., Wang Y.-X., Lippincott-Schwartz J. and Mattson M. P. (2015). Dendrosomatic Sonic Hedgehog signaling in hippocampal neurons regulates axon elongation. *J. Neurosci.* 35, 16126-16141. 10.1523/JNEUROSCI.1360-15.2015 26658865 PMC4682780

[BIO040592C46] Yao P. J., Manor U., Petralia R. S., Brose R. D., Wu R. T. Y., Ott C., Wangm Y.-X., Charnoff A., Lippincott-Schwartz J. and Mattson M. P. (2017). Sonic hedgehog pathway activation increases mitochondrial abundance and activity in hippocampal neurons. *Mol. Biol. Cell* 28, 387-395. 10.1091/mbc.e16-07-0553 27932496 PMC5341723

