# Supplementary material for: Sonic hedgehog expression in the postnatal brain
Source: Biol Open. 2019 Mar 5;8(3):bio040592. doi: 10.1242/bio.040592 (PMC6451348; doi:10.1242/bio.040592)
Supplement: Supplementary information [file biolopen-8-040592-s1.pdf]

Supplementary figures

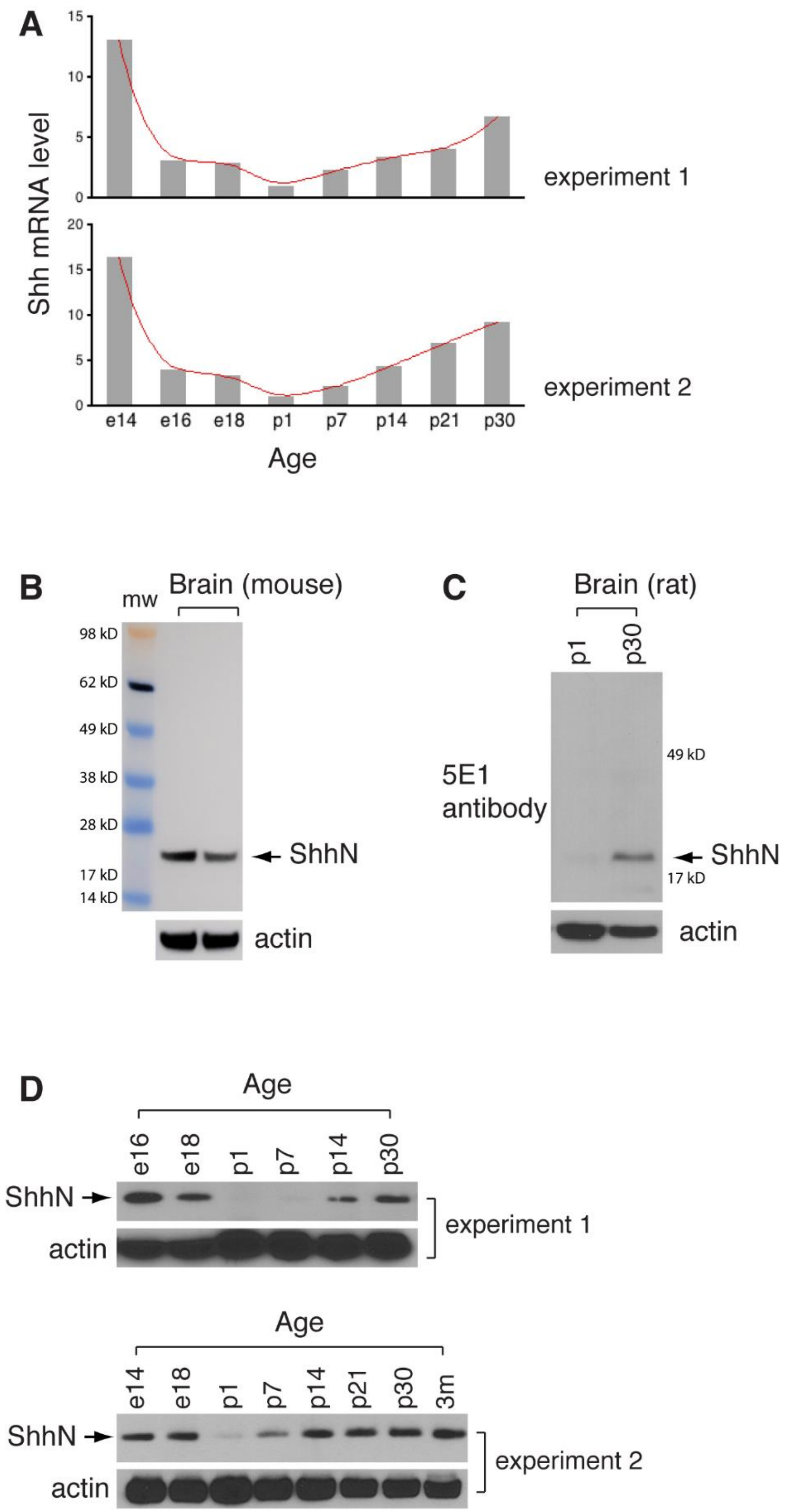

Biology Open • Supplementary information

Figure S1. Shh mRNA and protein expression levels in rodent brains during embryonic and postnatal development.

A, Quantitative RT-PCR assays of Shh mRNA level in rat cortex from embryonic day 14 (e14) to postnatal day 30 (p30). Shown are data from two experiments using a PCR primer set different from and in addition to the primer set used in Figure 1A.

B, Immunoblot with the ShhN antibody showing the ~19 kD ShhN as the main Shh species in mouse brain extracts (two different samples).

C, Immunoblot of rat brain extracts with a different ShhN antibody (5E1; Materials and Methods).

D, Additional examples of immunoblots profiling ShhN protein expression level in rat cortex from embryonic day 14 (e14) to postnatal day 30 (p30) or 3 months (3m).

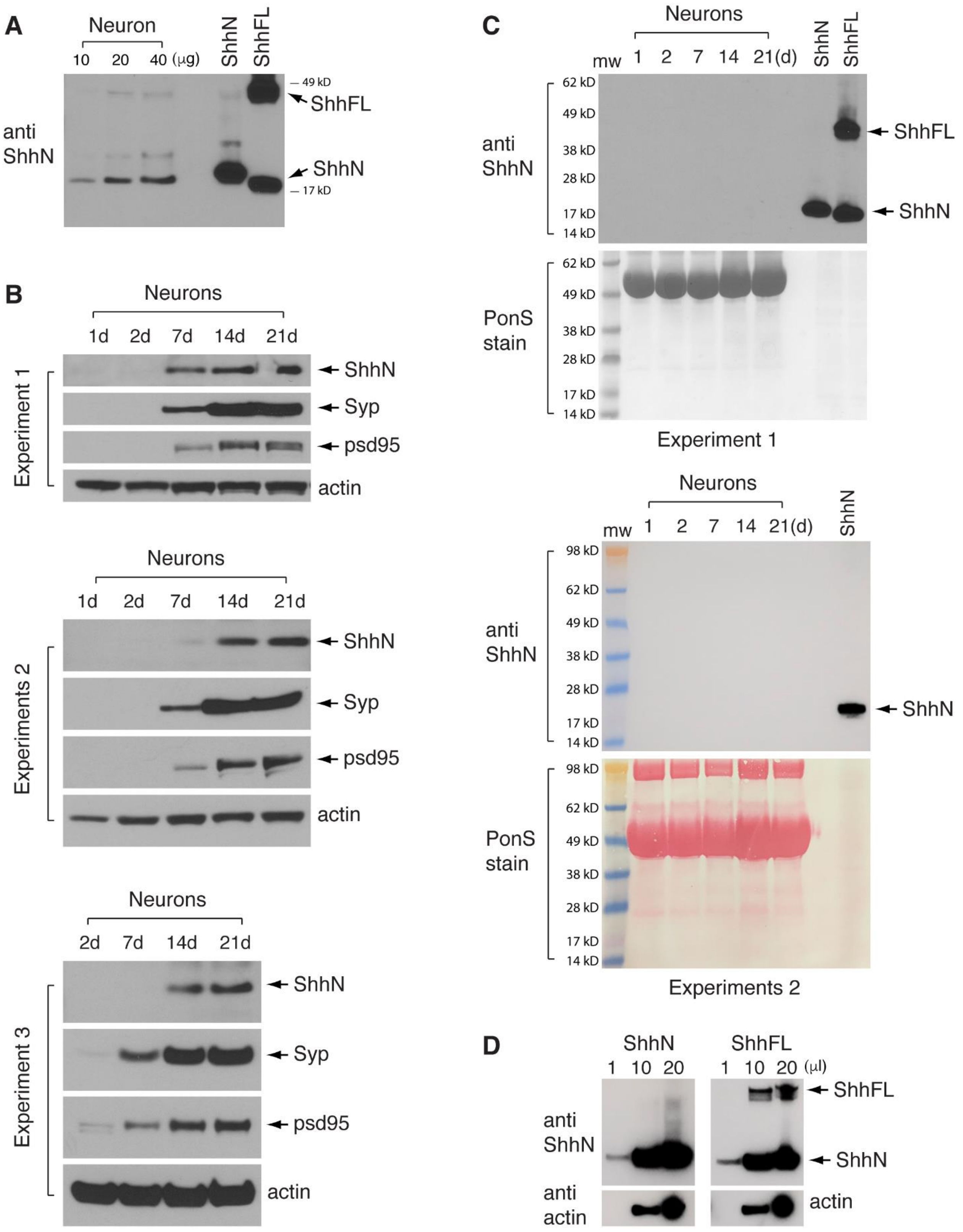

Biology Open • Supplementary information

Figure S2. ShhN protein expression levels in cultured hippocampal neurons.
A, Immunoblot of hippocampal neurons (21 days in culture) and HEK cells expressing N-terminal fragment of Shh (ShhN) or full-length Shh (ShhFL). In cultured hippocampal neurons, the ~19 kD ShhN is the most prominent protein band detected by the ShhN antibody and the intensity of the ~19 kD band correlates to the amount of total proteins.
B, Additional examples of immunoblots showing increased ShhN protein level in hippocampal neurons as these neurons matured in culture. d, days in culture.
C, Examples of immunoblots and corresponding Ponceau S-stained proteins showing undetectable extracellular ShhN protein in conditioned media from cultured hippocampal neurons.
D, Immunoblots of different amounts of the lysates from HEK cells expressing N-terminal fragment of Shh (ShhN) or full-length Shh (ShhFL). Blots show the titrations of the lysates in order to avoid overwhelming Shh protein band intensity as the result of overexpressed protein.

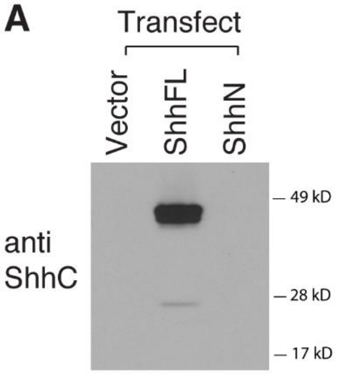

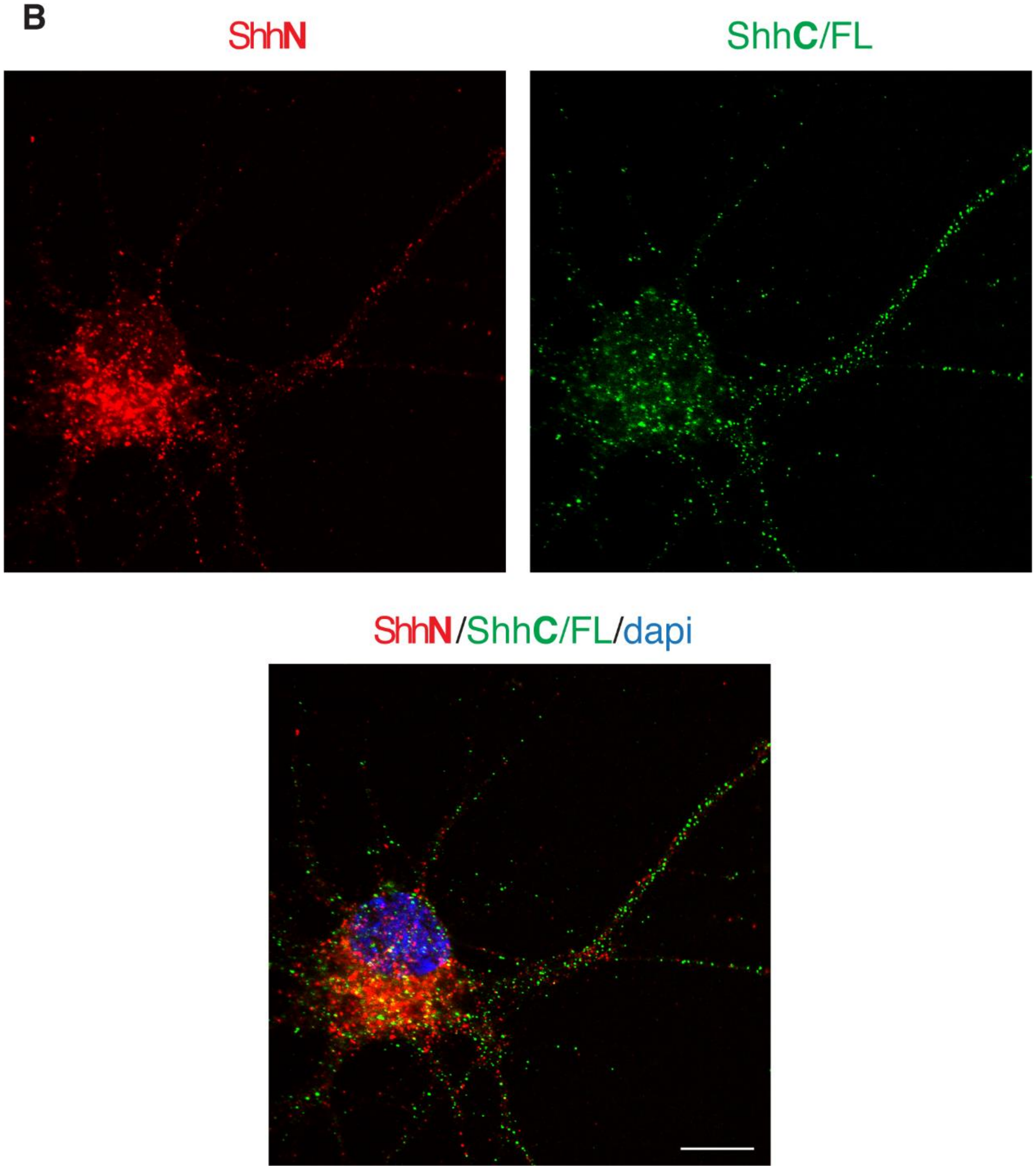

Biology Open • Supplementary information

Figure S3. ShhN and ShhC distribution in cultured hippocampal neurons.
A, Immunoblot shows the ShhC antibody detects full-length Shh (~45 kD) and a C-terminal Shh fragment (~25 kD). Samples are lysates of HEK cells expressing N-terminal fragment of Shh (ShhN)**,** full-length Shh (ShhFL) or vector.
B, Sample image of a hippocampal neuron showing a trend of higher ShhN (red) immunofluorescence labeling in soma (cell body) whereas a relatively even ShhC/FL (green) distribution throughout neurites. Scale bar, 10 μm.

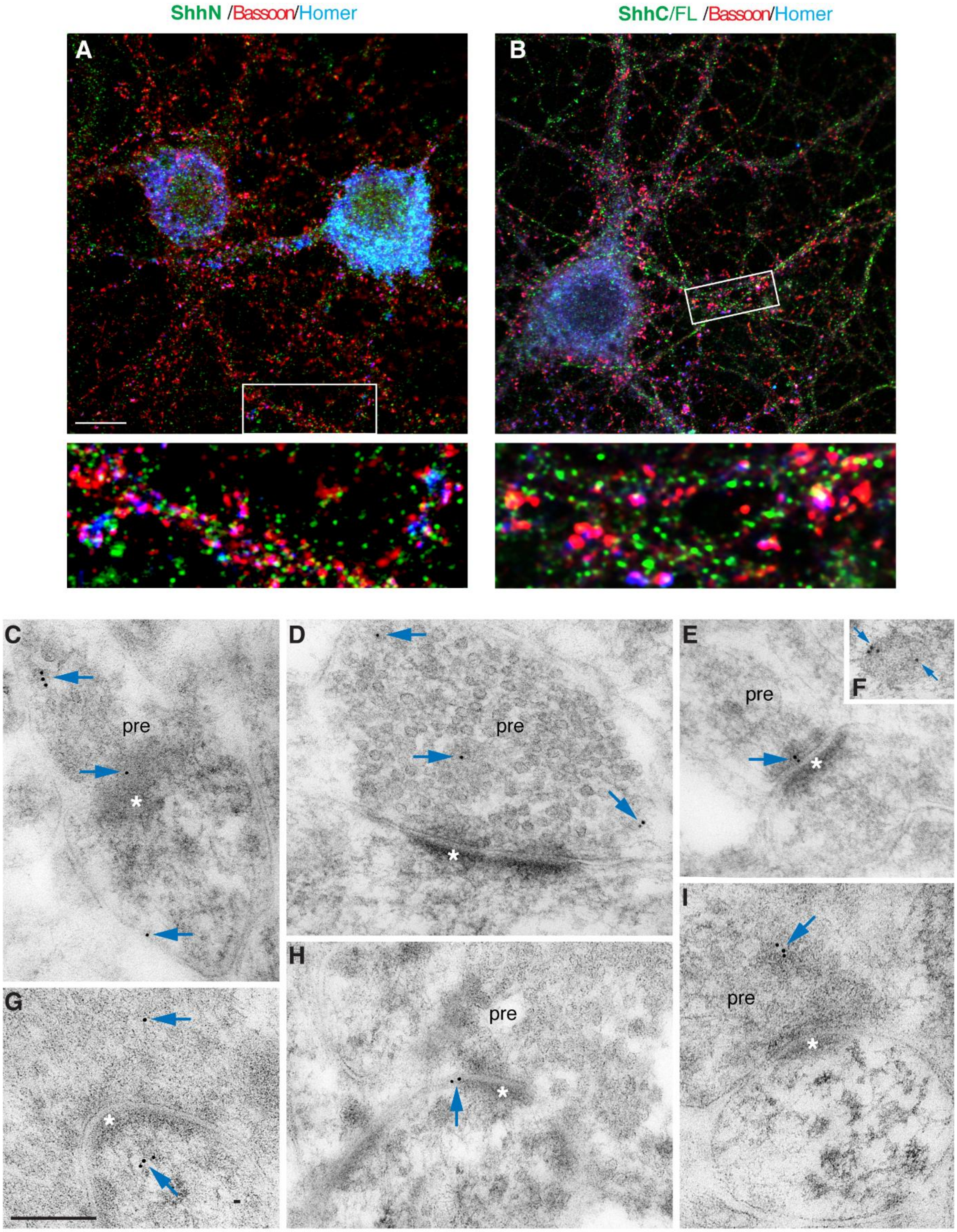

Biology Open • Supplementary information

Figure S4**.** ShhN and ShhC localization in synapses of hippocampal neurons.
A,***B***, Fluorescent images of mature hippocampal neuron (21 days in culture) co-labeled for ShhN (green in ***A***) or ShhC/FL (green in ***B***), a presynaptic marker, bassoon (red), and a postsynaptic marker, homer (blue).
C-***I***, Immunogold localization of ShhC in the CA1 stratum radiatum/pyramidale (***C***-***F***) or CA3 stratum lucidum (***G***-***I***) of the mouse hippocampus, with 10 nm immunogold (arrows). Labeling is found both in postsynaptic structures (***C,G,H***) and presynaptic terminal (pre; ***C,D,E,G,I***). Note localizations associated with the postsynaptic (***H***) or presynaptic (***E***) membrane. In ***F***, gold labels an endosome near the *trans*-Golgi network (*not shown*) in a neuron soma. Asterisk, postsynaptic density. Scale bar, 200 nm.

Biology Open • Supplementary information
